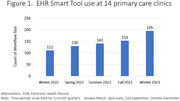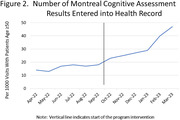# The role of primary care: An engagement study to improve evaluation of possible Alzheimer’s disease and related disorders at the entry point for patients in a large health system

**DOI:** 10.1002/alz.086149

**Published:** 2025-01-09

**Authors:** Barak Gaster, Jaqueline G. Raetz, Sarah McKiddy, Monica Zigman Suchsland, Amy P Hsu, Annette L. Fitzpatrick, Basia Belza, Joshua M. Liao

**Affiliations:** ^1^ University of Washington, Seattle, WA USA

## Abstract

**Background:**

The number of people with Alzheimer’s disease and related disorders (ADRD) is increasing. As a result, the role of primary care providers (PCPs) to detect ADRD is increasingly important. Early detection requires that PCPs are adequately trained to perform clinical evaluations, and health systems will require more knowledgeable PCPs to effectively collaborate with specialists to achieve the capacity needed to diagnose and manage ADRD.

**Method:**

With support of the Davos Alzheimer’s Collaborative, we developed a web‐training curriculum which was paired with tools integrated into the electronic health record (EHR). This intervention was implemented across a network of 14 community‐based primary care clinics from October 2022 to March 2023. PCPs in the network are primarily clinical, spending <5% of their time teaching or doing research. Patients in the network come from diverse backgrounds (41% non‐white). Data was extracted from the EHR to assess PCPs’ use of the tools and the number of cognitive evaluations performed. PCPs also received surveys measuring their confidence subscale for dementia.

**Result:**

All 153 PCPs in the network received access to the EHR tools. Their use of the tools gradually increased, rising to a frequency of 195 patient encounters in the final three‐months of the study period (Figure‐1). The number of cognitive tests entered into the EHR increased from 0.9 to 4.7 per 1000 patient visits >age 50 (p<0.01) (Figure‐2). Fifty PCPs completed the before and after training survey. Their mean reported ADRD‐related clinical confidence subscore increased from 2.54 to 3.74 (p<0.01).

**Conclusion:**

This PCP engagement intervention was effective when implemented across a large health system and was associated with more diagnostic cognitive evaluations and increased confidence in ADRD‐related ability by PCPs. As health systems experience growing pressures to meet the needs of patients with ADRD, they will increasingly require programs like this to educate and engage PCPs. This program is designed to be easy to disseminate to other health systems, a process currently underway via recorded trainings and downloaded EHR tools, in order to train PCPs to be both the initial points to begin evaluations and the effective continuity‐collaborators which health systems need.